# A Mechanistic Perspective on Plastically Flexible Coordination Polymers

**DOI:** 10.1002/anie.201914798

**Published:** 2020-02-04

**Authors:** Biswajit Bhattacharya, Adam A. L. Michalchuk, Dorothee Silbernagl, Max Rautenberg, Thomas Schmid, Torvid Feiler, Klaus Reimann, Ahmed Ghalgaoui, Heinz Sturm, Beate Paulus, Franziska Emmerling

**Affiliations:** ^1^ BAM Federal Institute for Materials Research and Testing Richard-Willstätter-Strasse 12489 Berlin Germany; ^2^ Max-Born-Institut für Nichtlineare Optik und Kurzzeitspektroskopie 12489 Berlin Germany; ^3^ Institut für Chemie und Biochemie Freie Universität Berlin Berlin Germany; ^4^ School of Analytical Sciences Adlershof (SALSA) Humboldt-Universität zu Berlin Berlin Germany

**Keywords:** coordination polymer, flexible crystals, mechanical properties, plastic deformation

## Abstract

Mechanical flexibility in single crystals of covalently bound materials is a fascinating and poorly understood phenomenon. We present here the first example of a plastically flexible one‐dimensional (1D) coordination polymer. The compound [Zn(μ‐Cl)_2_(3,5‐dichloropyridine)_2_]_*n*_ is flexible over two crystallographic faces. Remarkably, the single crystal remains intact when bent to 180°. A combination of microscopy, diffraction, and spectroscopic studies have been used to probe the structural response of the crystal lattice to mechanical bending. Deformation of the covalent polymer chains does not appear to be responsible for the observed macroscopic bending. Instead, our results suggest that mechanical bending occurs by displacement of the coordination polymer chains. Based on experimental and theoretical evidence, we propose a new model for mechanical flexibility in 1D coordination polymers. Moreover, our calculations propose a cause of the different mechanical properties of this compound and a structurally similar elastic material.

## Graphical abstract

**Bending the rules**: The first example of a plastically flexible 1D coordination polymer is described. [Zn(μ‐Cl)_2_(3,5‐Cl_2_Py)_2_]_*n*_ is flexible over two crystallographic faces and a single crystal remains intact when bent to 180°. Displacement rather than deformation of the coordination polymer chains appears to be responsible for the observed macroscopic bending.

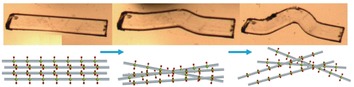

Highly ordered materials which combine organic and metal species have surged to the forefront of modern materials sciences.^1^ These hybrid materials have been identified as promising candidates in gas separation, storage, sensing, catalysis, drug delivery, and as proton‐conducting fuel cells.^1^, ^2^ There is particular interest in metal‐organic hybrid materials with 1D or 2D structures.^3^ This is a result of their unique and tunable electronic and magnetic properties as well as their considerable potential for applications as advanced functional materials.^4^ Some of these metal‐organic hybrid materials have also shown metal‐like electrical conductivity.^5^ Single crystals of these materials are typically brittle, thereby restricting their use in practical applications as flexible electronic, magnetic, or optical devices.

Advances in molecular crystal science has revealed the existence of plastically and elastically flexible single‐crystal systems.^6^, ^7^, ^8^ Elastically flexible materials have been proposed for application as reversible stress sensors,^7^ while plastically flexible materials offer a route to moldable devices.^8^ Hence, these materials combine the excellent physicochemical properties of highly ordered materials with enhanced durability for a greater range of applications. Only recently has mechanical flexibility been identified in 1D coordination polymers (CPs).^9^ All reported crystals of 1D CPs have fractured after small elastic deformations, without exhibiting any notable plastic regime. The existence of plastically deformable single‐crystal 1D CPs has not been demonstrated.

Herein, we report the first example of a single‐crystal 1D CP with extensive mechanical plasticity: [Zn(μ‐Cl)_2_(3,5‐dichloropyridine)_2_]_*n*_ (**1**; Figure 1). Although the crystal structure of **1** was described previously, the mechanical properties have not yet been examined.^10^ **1** crystallises in the tetragonal space group *P* $\bar{4}$ *b*2 and contains a 1D CP chain along the crystallographic *c*‐axis. These CP chains are based on chloride‐bridged edge‐sharing zinc(II) octahedra (Figure 2). Further description of the crystallographic structure is given in the Supporting Information (Figures S1–S3, Tables S1 and S2). The qualitative mechanical properties of single crystals of **1** were examined by the established three‐point bending method (Figures 1 c–k).^7^, ^8^ When stressed perpendicular to the CP chains, that is, the long crystallographic faces ($\bar{1}$ 10) and ($\bar{1}$ $\bar{1}$ 0), the crystals bent without visible fracture (Figures 1 c–h and see Videos S1 and S2 in the Supporting Information). Remarkably, **1** could be readily bent to acute angles without macroscopic fracture (Figures 1 e,h). This mechanical behaviour is different from that of all previously reported mechanically flexible 1D CP crystals.^9^ The bending in both directions was irreversible (plastic) over the duration of testing and remained upon withdrawal of the deforming force. In contrast, when crystals were stressed along the (001) face (i.e. parallel to the CP chains), brittle fracture was observed (Figures 1 i–k, and Video S3). Hence, **1** exhibits 2D plasticity as a result of the crystallographic symmetry, with the molecular packing identical along both crystal faces.

**Figure 1:**
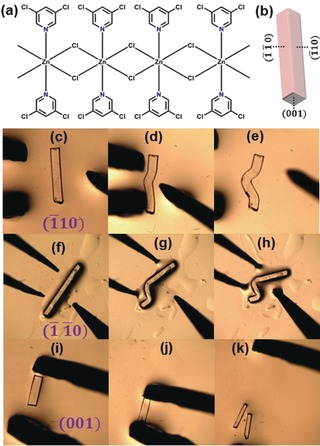
a) Chemical structure of [Zn(μ‐Cl)_2_(3,5‐dichloropyridine)_2_]_*n*_ (**1**). b) Crystal face indices of a crystal of **1** determined by single‐crystal X‐ray diffraction. c–k) Optical microscopic photographs of single crystals of **1** undergoing three‐point bending. Tweezers were used to restrain the crystal ends and a needle exerted a force between the restrained ends. Plastic bending along the major crystallographic faces (c–e) for ($\bar{1}$ 10) and (f–h) for ($\bar{1}$ $\bar{1}$ 0). i–k) Brittle fracture when stressed along the (001) crystallographic face.

**Figure 2:**
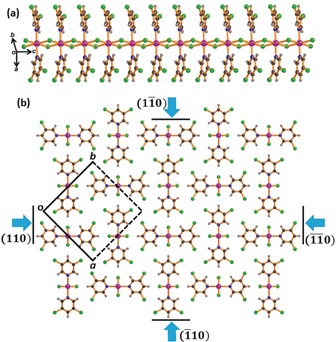
Crystal structure of the coordination polymer Zn(μ‐Cl)_2_(3,5‐Cl_2_Py)_2_]_*n*_ (**1**). a) Structure of the covalently bonded coordination polymer chain. b) Crystallographic packing of the adjacent polymeric chains along the [001] plane. Black lines represent the bending faces (110), ($\bar{1}$ $\bar{1}$ 0), (1$\bar{1}$ 0), and ($\bar{1}$ 10). Cyan arrows indicate the directions in which the mechanical forces are applied. The box displays the crystallographic unit cell.

The molecular packing arrangement of **1** is very similar to those of previously reported elastically flexible CP crystals.^9^ Understanding the origin of the different mechanical behaviour of **1** is, therefore, of fundamental interest. To examine the effects of mechanical bending on the integrity of the crystal lattice of **1**, we quantified the mechanical properties of its plastically flexible faces by atomic force microscopy (AFM).^11^ AFM force–distance curves (FDCs) were measured on the ($\bar{1}$ 10) face of **1**. A Young's modulus of *E=*26 GPa (Poisson's ratio of *ν*=0.28) was obtained for straight crystals (Figures S4) and this is well‐supported by DFT calculations (*E=*27.8 GPa and *ν*=0.28). These mechanical properties demonstrate that **1** is notably stiffer than most organic plastic molecular crystals (typical values of *E*≈0.1–10.0 GPa).^12^ As a consequence of the bulk plasticity of **1**, the single crystal did not recover fully when the force was removed. Instead, a small hysteresis was observed in the FDCs (Figure 3, red and Figure S5).

**Figure 3:**
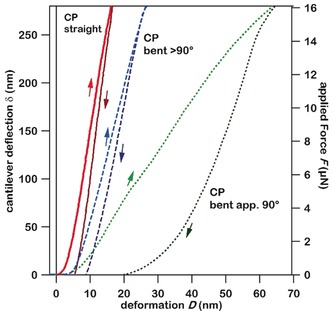
AFM measurement of deflection/force versus deformation of **1**. The solid vertical line at *D*=0 denotes contact. Data are shown for loading and unloading, as indicated by arrows. All curves shown are average curves of at least 30 measurements. The curves were measured on non‐bent (solid), bent >90° (dashed), and bent ca. 90° (dotted) parts. The hysteresis values *w*_h_ calculated from averaged force‐deformation curves are *w*_h_≈5 fJ for the unbent crystals, *w*_h_≈7.6 fJ for >90° bent crystals, and *w*_h_≈203 fJ for ca. 90° bent crystals.

FDCs were next measured in the curvature of bent single crystals of **1**. When crystals were bent to angles >90°, the material stiffness (indicated by the FDC slope) and hysteresis remained virtually unchanged compared with the parent crystal (Figure 3, blue). The crystalline lattice, therefore, remained largely unperturbed by the mechanical stress. Microfocus X‐ray diffraction verified the preserved crystallinity (Figure S6), although a slight broadening of the Bragg reflections indicates partial loss of long‐range translational symmetry (i.e. mosaicity).^13^ FDCs collected on crystals bent to about 90° showed they were more compliant than the straight crystals (Figure 3, green). Moreover, a marked increase in the hysteresis of the FDCs was observed.^13^ Despite remaining macroscopically intact, it seems that long‐range order was compromised at large deformations. This was not observed in the corresponding microfocus X‐ray diffraction collected from this region, which showed Bragg scattering consistent with a highly polycrystalline material (Figure S6).

Although the AFM studies indicated the overall crystalline lattice remained intact upon bending, it remained unclear whether the mechanical stress accumulated in or between the CP chains. To investigate the effects of bending on the molecular structure, we compared the Raman spectrum of a straight crystal of **1** to those obtained at various locations in bent (ca. 90°) crystals of **1** (Figure 4 a). Surprisingly, no detectable shifts were observed in the Raman spectra upon mechanical deformation (Figure 4 a; see also Figures S7 and S8 as well as Table S3). This finding strongly implies that the flexibility of **1** does not result from bending or rupture of the covalent CP network. We, therefore, explored the effects of bending on the vibrations of the crystal lattice by terahertz time domain (THz‐TD) spectroscopy (Figure 4 b). All but two of the low‐frequency bands (<3 THz, 100 cm^−1^) were lost in the THz‐TD spectrum upon bending. The two remaining bands (between ca. 33 and 50 cm^−1^) correspond to rotation and translation of the aromatic CP ligands, as indicated by DFT calculations (see Figure S9 and Table S4). A lack of well‐defined external vibrational modes is a common feature of nanocrystalline or amorphous materials. THz‐TD spectroscopy, therefore, indicates a loss of long‐range order when **1** is bent to about 90°, consistent with the AFM studies.

**Figure 4:**
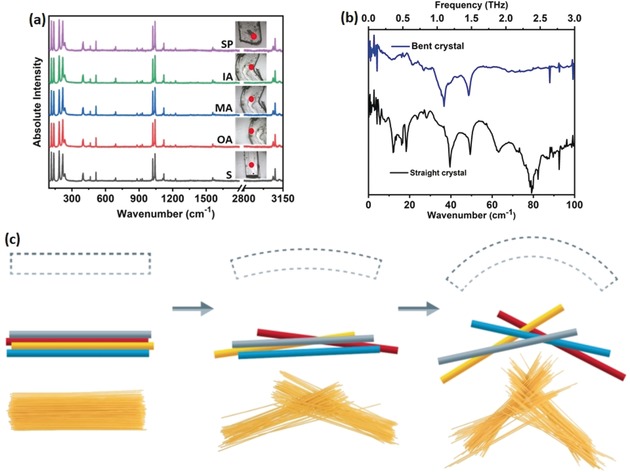
Vibrational spectra of linear and bent **1**. a) Unpolarised Raman spectra collected at different locations in the crystal at different stages of plastic bending. The Raman spectra are displayed alongside the corresponding crystal, and the laser spot is indicated as a red dot. The crystals are denoted as: s: straight; OA: outer arc; MA: middle arc; IA: inner arc; and SP: straight part of a bent crystal. b) THz‐TD (time domain) spectra collected for the ca. 90° bent crystal (blue) and for a straight crystal (black). c) Schematic representation of the spaghetti model for the bending of the coordination polymer. Note that each bundle of straws represents a cluster of CP chains.

The experimental data suggested that **1** retained its mechanical properties and crystallinity upon bending to shallow angles. When the bending is extended to acute angles, the bulk order is largely lost, and only small coherent domains persist.13b As evidenced from spectroscopic measurements, the stress of bending does not accumulate within the CP chains, but between them. Typically, the plasticity of molecular crystals is ascribed to the presence of slip planes. However, no such features are present in **1**. We, therefore, propose that the flexibility of CPs is based on a spaghetti model (Figure 4 c). Here, the initial stress leads to slippage of CP chains perpendicular to the bend. Concurrently, clusters of CP chains are displaced parallel to the bend and become interwoven. This leads to the formation of a broad distribution of small‐angle grain boundaries throughout the structure. Hence, our model accounts for the loss of long‐range crystallinity and the formation of a powder‐like structure.

The principal deformation of this model requires the breaking of interchain CP⋅⋅⋅CP interactions. To explore this model further, we calculated the potential energy surface (PES) associated with this deformation of **1** (Figure 5 a; see also Figures S10 and S11). The calculated PES was compared with that of an elastically flexible CP reported by Đaković et al.: [Cd(μ‐Br)_2_(2‐chloropyrazine)_2_]_*n*_ (**2**).^9^

**Figure 5:**
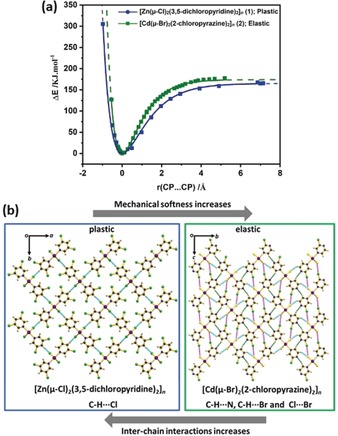
a) Potential energy curves for **1** (blue) and **2** (green)^9^ as a function of the isotropic expansion of the CP⋅⋅⋅CP chains. The value of *r*(CP⋅⋅⋅CP) corresponds to the equilibrium normalized metal⋅⋅⋅metal distances. The solid lines are fits to a conventional Morse potential. b) Molecular packing diagram along the CP axis of **1** and **2**. The CP⋅⋅⋅CP interactions are highlighted as: magenta (halogen⋅⋅⋅halogen), green (C−H⋅⋅⋅N), and cyan (C−H⋅⋅⋅Br for **2** and C−H⋅⋅⋅Cl for **1**).

Within our spaghetti model, plastic deformation requires stable interweaving of the displaced CP network. Hence, plastic deformation is favoured in cases where inter‐CP displacements have low energy penalties. The PES corresponding to the isotropic expansion of the inter‐CP crystallographic plane was obtained for **1** and **2**. A fit to the conventional Morse potential thus provides access to a force constant *k*_CP_ associated with the necessary perturbation of the unit cell (Figure 5 a; see also Figures S10 and S11). The *k*_CP_ value calculated for the plastically flexible compound **1**, *k*_CP_=255.01 kJ Å^−2^, is approximately half the value calculated for the elastically flexible compound **2**, *k*_CP_=431.17 kJ Å^−2^ (Table S5). This magnitude is equivalent to the difference in the force constants for C−C versus C=C covalent bonds. Hence, the elastically flexible crystals exhibit substantially stronger restorative forces than the plastic CP. It follows that any deformation of the crystalline lattice of **2** is less likely to persist, and relaxation of the higher energy deformed structures of **2** to their unperturbed, equilibrium structures will be favoured.

From the perspective of material design, the markedly different values of *k*_CP_ obtained for the plastic and elastic crystals is derived from their different inter‐CP interactions. In the case of **2**, a series of “strong” interactions such as, halogen⋅⋅⋅halogen, C−H⋅⋅⋅N, and C−H⋅⋅⋅Br interactions are present (Figure 5 b; see also Figures S13 and S14). Only very weak C−H⋅⋅⋅Cl interactions are present within the structure of **1** (Figure 5 b; see also Figures S12 and S14). In the crystal structure of **1**, the lack of strong interchain interactions facilitates the formation of stable interwoven networks as a result of mechanical deformation. In contrast, the relatively strong halogen⋅⋅⋅halogen and C−H⋅⋅⋅N interactions in the elastic crystal of **2** favour the non‐deformed crystalline structure, as evidenced by the large *k*_CP_ value. Hence, these strong interactions do not permit the formation of stable interwoven networks and restore the pristine crystalline geometry upon removal of the deforming force.

Our proposed spaghetti model represents an idealised view of mechanical flexibility in 1D CPs. Notably, our model does not yet include an understanding of the CP structure at the ends of the macroscopically bent crystal. Moreover, a deeper understanding of CP chain termination inside the bent crystal is required. Further developments will permit a more rigorous understanding of these remarkable mechanically flexible 1D materials.

In summary, we report here the first example of a 1D CP crystal that exhibits predominantly plastic mechanical flexibility. As a consequence of the crystal symmetry, two of the three faces are mechanically flexible. Our studies show that the mechanical flexibility of the material correlates to the internal atomistic structure. Detailed AFM and microfocus X‐ray diffraction measurements show that the single crystallinity remains largely unchanged at small deformations. However, large mechanical deformations lead to a drastic failure of the crystal‐lattice structure. We show that these compromised mechanical properties result from the formation of a highly polycrystalline phase at the site of mechanical perturbation. This phenomenon is further supported by micro‐Raman and THz‐TD spectroscopy. Well‐known examples of plastically bendable molecular crystals are typically explained on the basis of a slip‐plane model. However, the flexible CPs do not contain the necessary slip planes required for the established model. We, therefore, proposed a new model to rationalise the mechanically flexibility observed in the single‐crystal 1D CP: the spaghetti model. This model requires the formation of interwoven networks of CPs upon bending, and hence separation of the CP chains. DFT studies indicate that the separation of the CP chains occurs more readily in the plastically bendable crystal. In contrast, the same deformation in an elastic crystal is associated with large energy penalties. Hence, the pristine crystalline geometry of elastic crystals is readily restored upon removal of the deforming force. The insight obtained through experimental and theoretical studies has allowed us to propose a new mechanism which has been applied to two independent systems. We expect our findings to encourage further explorations to demonstrate the universal applicability of our model.

## Conflict of interest

The authors declare no conflict of interest.

## Supporting information

As a service to our authors and readers, this journal provides supporting information supplied by the authors. Such materials are peer reviewed and may be re‐organized for online delivery, but are not copy‐edited or typeset. Technical support issues arising from supporting information (other than missing files) should be addressed to the authors.

Supplementary

Supplementary

Supplementary

Supplementary

Supplementary

Supplementary

Supplementary
