# Supplementary material for: A Mechanistic Perspective on Plastically Flexible Coordination Polymers
Source: Angew Chem Int Ed Engl. 2020 Feb 4;59(14):5557–61. doi: 10.1002/anie.201914798 (PMC7155097; doi:10.1002/anie.201914798)
Supplement: Supplementary file 1 — Supplementary [file ANIE-59-5557-s001.pdf]

Angewandte Chemie
Eine Zeitschrift der Gesellschaft Deutscher Chemiker

Supporting Information

A Mechanistic Perspective on Plastically Flexible Coordination Polymers

*Biswajit Bhattacharya[+], Adam A. L. Michalchuk[+], Dorothee Silbernagl, Max Rautenberg, Thomas Schmid, Torvid Feiler, Klaus Reimann, Ahmed Ghalgaoui, Heinz Sturm, Beate Paulus, and Franziska Emmerling**

anie_201914798_sm_miscellaneous_information.pdf
anie_201914798_sm_Video_S1.mp4
anie_201914798_sm_Video_S2.mp4
anie_201914798_sm_Video_S3.mp4

Abstract: Mechanical flexibility in single crystals of covalently bound materials is a fascinating and poorly understood phenomenon. We present here the first example of a plastically-flexible one dimensional (1D) coordination polymer. The compound $[Zn(\mu\text{-}Cl)_2(3,5\text{-}Cl_2Py)_2]_n$, is flexible over two crystallographic faces. Remarkably, the single crystal remains intact when bent to 180o. Through the combination of microscopy, diffraction, and spectroscopic studies we probe the structural response of the crystal lattice to mechanical bending. Deformation of the covalent polymer chains does not appear to be responsible for the observed macroscopic bending. Instead, our results suggest that mechanical bending occurs by displacement of the coordination polymer chains. Based on experimental and theoretical evidence, we propose a new model for mechanical flexibility in 1D coordination polymers. Moreover, our calculations propose an origin for the different mechanical properties of this compound and a structurally similar elastic material. The results presented here offer novel insight into the mechanism of mechanical flexibility in coordination polymer crystals and the potential of tuning their physical properties by mechanical force.

DOI: 10.1002/anie.2016XXXXX

Table of Contents

**S1. Experimental Details**	**S3-S4**
**S2. Computational Details**	**S5**
**S3. Crystal Structure Description**	**S6-S8**
**S4. Characterization of straight and bent crystal of $[Zn(\mu\text{-}Cl)_2(3,5\text{-dichloropyridine})_2]_n$**	**S9-S15**
***S4.1 Atomic Force Microscopy*.**	**S9-S11**
S4.2 Micro-focus Single crystal X-ray diffraction	**S12**
S4.3 Raman spectroscopy	**S13-S15**
**S5. Computational Vibrational Calculations**	**S16-S17**

**S6. Analysis of Coordination Polymer Potential Energy Surfaces**	**S18-S19**
**S7. Qualitative Analysis of Electronic Structure**	**S20-S21**
**S8. Intermolecular Interactions**	**S22**
**S9. PXRD and ATR-IR spectroscopy**	**S23**
**S10. References**	**S24**

S1. Experimental Details

S1.1 Materials.

Zinc(II) chloride (99.999%; CAS No: 7646-85-7) and 3,5-dichloropyridine (98%; CAS No: 2457-47-8) were purchased from Sigma-Aldrich and used as received. Ethanol (99%, CHEMSOLUTE) was of AR grade and used as received.

S1.2 Physical Measurements.

Elemental analyses (carbon, hydrogen, and nitrogen) were performed using a Perkin-Elmer 240C elemental analyzer. Attenuated total reflection-infrared (ATR-IR) spectra were recorded using a Nicolet FT-IR Nexus spectrometer by averaging 100 scans at a resolution of 2 cm^{-1}. Powder X-ray diffraction (PXRD) data were collected on a Bruker D8 diffractometer with Cu-Kα1 radiation (λ = 1.54106 Å) in a range of 5.0° ≤ 2 θ ≤ 40°. The data were obtained in transmission mode with a step size of 0.009°.

S1.3 Preparation of $[Zn(\mu\text{-}Cl)_2(3,5\text{-dichloropyridine})_2]_n$ (1).

Zinc(II)chloride (2 mmol, 0.273 g) was dissolved in 20 mL ethanol and 3,5-dichloropyridine (4 mmol, 0.592 g) was dissolved in 20 mL of ethanol. The resulting colorless solutions were mixed together with constant stirring for 3 hours. After seven days, colorless block shaped crystals were obtained from the filtrate. Yield 85%. Anal. Calc. for $C_{10}H_6Cl_6N_2Zn$ (MW: 432.26): C, 27.79; H, 1.40; N, 6.48%. Found: C, 27.35; H, 1.29; N, 6.36%.

S1.4. Single crystal X-ray diffraction.

Single crystal XRD data of crystals, individually mounted on a glass tip, will be collected on a Bruker D8 Venture system with graphite-monochromatic Mo Kα radiation (λ = 0.71073 Å). Data reduction was performed with Bruker AXS SAINT[1] and SADABS[2] packages. The structure was solved by SHELXS 2018[3] using direct method and followed by successive Fourier and difference Fourier synthesis. Full matrix least-squares refinements were performed on F^2 using SHELXL-2018[3] with anisotropic displacement parameters for all non-hydrogen atoms. All other calculations were carried out using SHELXS 2018,[3] SHELXL 2018,[3] and WinGX (Ver-1.80),[4] whereas Mercury v3.6[5] is used to visualize and to draw some of the figures for the structures. Detailed data collection strategy and structure refinement parameters along with crystallographic data are presented in Table S1.

S1.5. Atomic Force Microscopy.

Atomic Force Microscopy (AFM) measurements were carried out with an MFP-3D microscope (Asylum Research, Oxford Instruments, High Wycombe, UK). As AFM probe a Pointprobe PPP-NCHR (Nanosensors, Neuchâtel, Switzerland) was used.

S1.6 Raman Spectroscopy

Raman spectroscopy was performed by employing a Horiba Jobin Yvon Labram HR800 Raman microscopy system, which includes an Olympus BX41 microscope. The white-light microscopic imaging part of the system was used in conjunction with a 10×/N.A. = 0.25 objective lens (with N.A. denoting the numerical aperture) to acquire images of the crystals (insets of Figure 3 in the main text) and select measurement spots. For Raman measurements, the light emitted by a continuous-wave diode-pumped solid-state laser having a wavelength of 532 nm was focused onto the sample surface by a 50×/N.A. = 0.75 objective, and the scattered light was collected by the same lens. Reflected light and Rayleigh scatter were rejected by a bandpass filter, and the Stokes-shifted Raman-scattered light was dispersed by a 1800-mm^{-1} grating in a spectrograph and detected by a liquid-nitrogen-cooled charge-coupled device (CCD) camera operated at -130°C. The spectrometer entrance slit was 100 µm wide and the confocal pinhole was in the fully open position (1000 µm). In this configuration, the spectral resolution varied between 0.5 cm^{-1} per CCD pixel (at 100 cm^{-1} Raman shift) and 0.3 cm^{-1} per CCD pixel (at 3800 cm^{-1} Raman shift) within the observed spectral range. The spatial resolution of the system mainly depends on the numerical aperture and the laser wavelength. The lateral laser spot diameter is approx. 1 µm in this configuration, while the depth resolution is roughly 20 µm.[6]

S1.7 Time Domain THz Spectroscopy (TD-THz)

The THz measurements were performed with a commercial THz time domain spectrometer (TERA K15, Menlo Systems), which works in the spectral range from 0.1 to 5 THz (3-170 cm^{-1}) with a spectral resolution of 0.01 THz (0.3 cm^{-1}). The THz radiation in this spectrometer is linearly polarized. Thus, polarization-dependent measurements are made by rotating the sample around its axis so that the direction of the THz electric field is either along or perpendicular to the sample axis. The sample is mounted on a metal plate with a 0.5 mm diameter hole to ensure that no THz radiation is going through the sample.

S2. Computational Details

S2.1. Periodic Plane Wave Density Functional Theory

All plane wave Density Functional Theory (DFT) calculations were performed within the Quantum ESPRESSO v6.4 suite.[7] Input crystal structures were taken from experimental data, either obtained in this work $[Zn(\mu\text{-}Cl)_2(3,5\text{-dichloropyridine})_2]_n$ or from the Cambridge Crystallographic Database (CCDC): REF QAWGON.[8] Calculations were performed using the exchange-correlation functional of Perdew-Burke-Erzenhof (PBE)[9] with the Grimme D3 van der Waals correction, without damping.[10] Ultrasoft pseudopotentials were used throughout, and the wave function was expanded in plane waves to a kinetic energy cutoff of 60 Ry (charge density cut-off of 600 Ry). Atomic positions and unit cell parameters were fully relaxed until residual atomic forces < 10^{-4} Ry.$Bohr^{-1}$, and a total unit cell stress of < 0.3 kbar was obtained. SCF convergence was accepted < 10^{-8} Ry. The electronic structure was sampled on a Monkhorst-Pack *k*-point grid of size 4 x 1 x 1.[11]

S2.2. Phonon frequency calculations

The calculation of vibrational frequencies and eigenvectors was performed using the Phonopy v2.4.1 suite.[12] As only Brillouin zone centre frequencies were calculated, displacements were generated over a primitive cell representation. The acoustic sum rule was corrected analytically. Correction for LO-TO splitting was not included.

S2.3. Electronic Band Structures and Crystal Orbital Hamilton Population Analysis

The electronic band structures for each system were calculated at the relaxed geometry, as outlined in Section S2.1. The band structures were derived across high symmetry lines in the Brillouin zone, as suggested by SeeKPath.[13] COHP analysis was performed on the same geometry as implemented in the Lobster v3.2 software.[14] Due to computational requirements, however, the electronic structure was calculated using Plane Augmented Wave pseudopotentials. In both cases, the default basis sets proved suitable when included s, p, and d functions in the valence projection. Both systems resulted in an absolute charge spilling parameter < 1.1%.

S2.4. Electrostatic Potential Maps

The electrostatic potential maps were calculated on isolated CP chains. Each CP chain was extended across three-unit cells and truncated across the halogen-metal bonds to conserve electroneutrality. Calculations were performed using Orca v4.2 at the B3LYP/def2-svp level.[15] The molecular electrostatic maps were generated on a 50 x 50 x 50 grid.

S3. Crystal Structure Description

[Zn(μ-Cl)$_2$(3,5-dichloropyridine)$_2$]$_n$: This CP crystallises in the tetragonal space group ($P\bar{4}b2$). The metal centers are located in the special position with hexa-coordination nearly perfect octahedral geometry (Figure S1). The equatorial plane of the octahedron is created by four symmetry related chlorine atoms; whereas two symmetry related nitrogen atoms of 3,5-dichloropyridine ligands occupy the axial position (Figure S1). In the 1D chains, the chloride bridged Zn(II) octahedra extend along direction [001], with Zn⋯Zn separation of 3.6510(2) Å (Figure 2, Table S2). Adjacent chains are assembled in an anti-parallel fashion, with all of faces of the crystal being stabilized C-H⋯Cl (2.84 Å) interactions (Figure S2-S3).

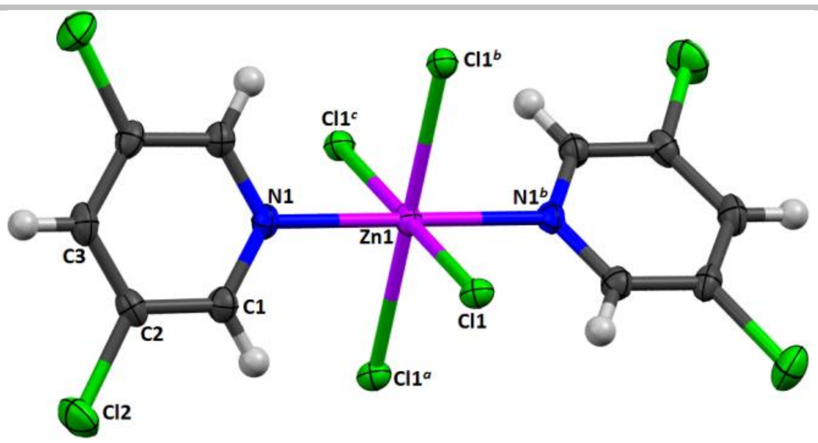

Figure S1. Thermal ellipsoid drawing of the asymmetric unit of [Zn(μ-Cl)$_2$(3,5-dichloropyridine)$_2$]$_n$ (**1**) showing atom labels and 50% probability of ellipsoids.

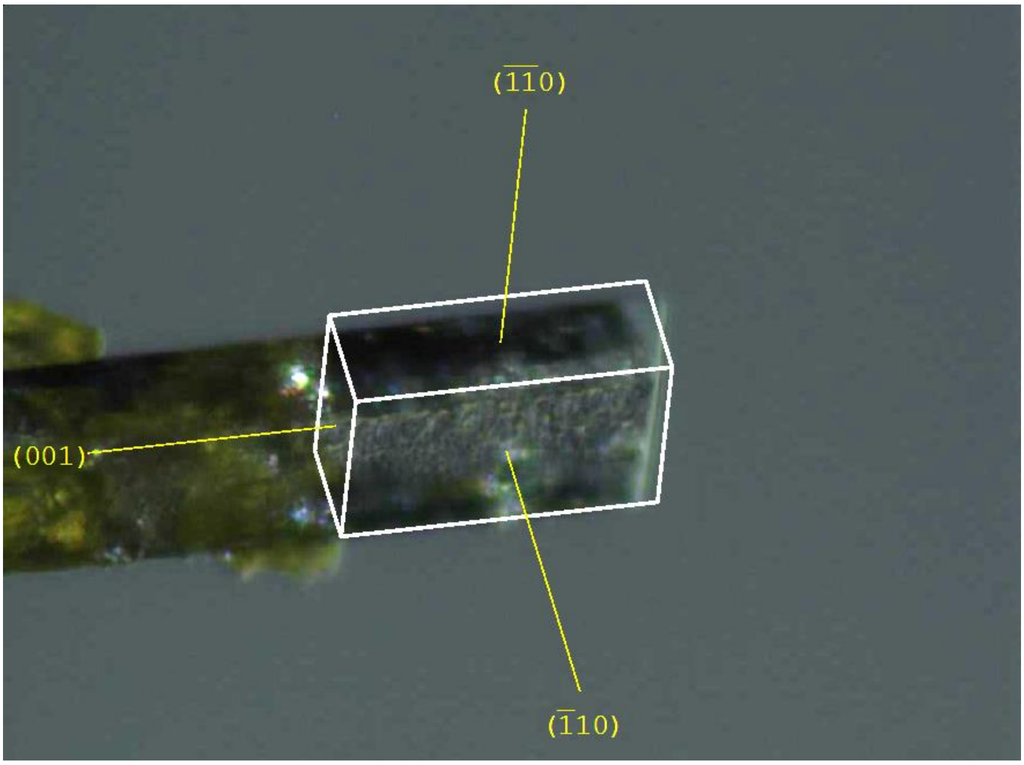

Figure S2. Face indexed image of pristine crystal of [Zn(μ-Cl)$_2$(3,5-dichloropyridine)$_2$]$_n$ (**1**).

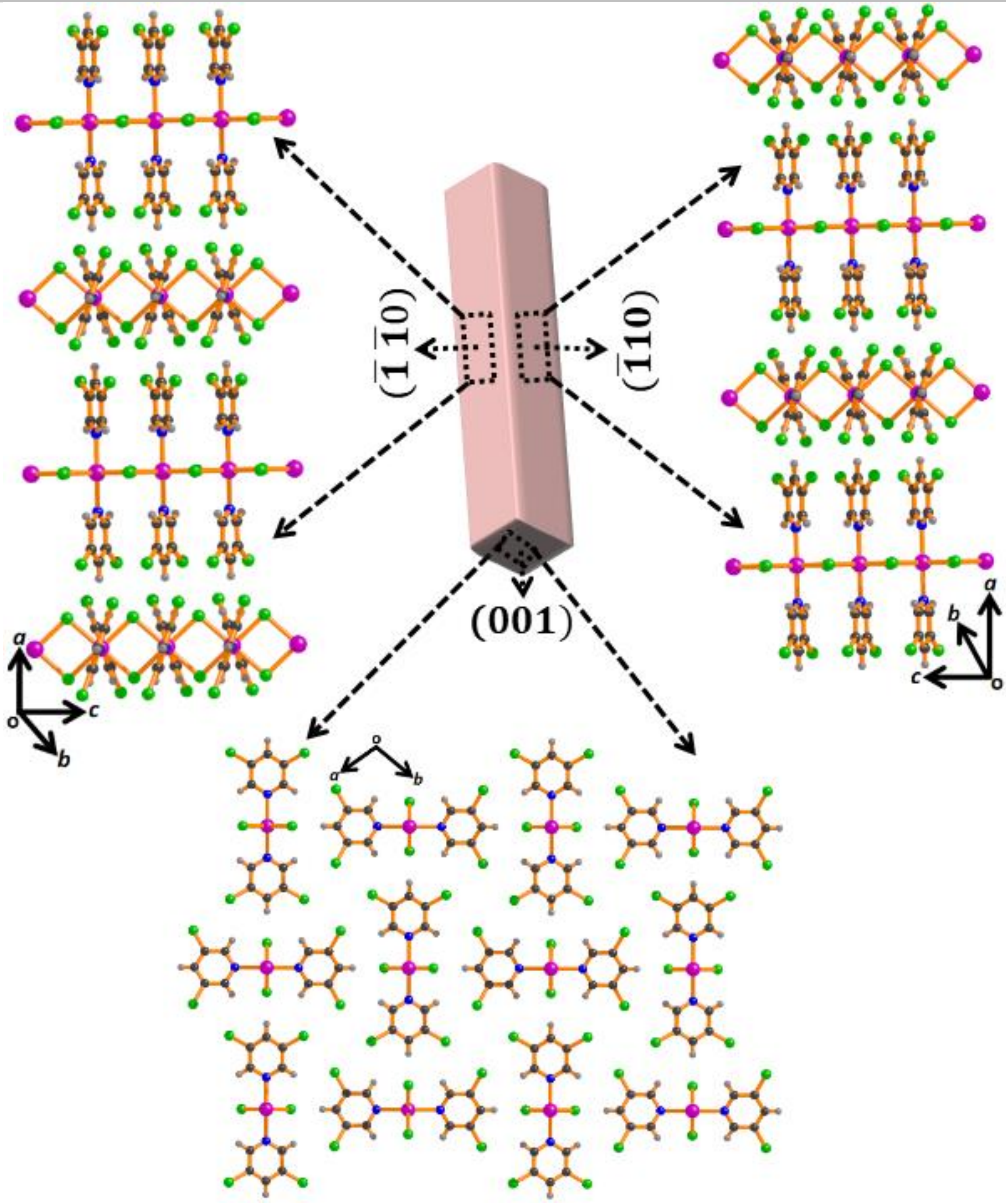

Figure S3. Crystallographic packing of [Zn(μ-Cl)$_2$(3,5-dichloropyridine)$_2$]$_n$ (**1**) according to BFDH algorithm, as viewed along crystal faces. Crystal faces are indexed by single crystal diffraction.

Table S1. Crystallographic and Structural Refinement Parameters of [Zn(μ-Cl)$_2$(3,5-dichloropyridine)$_2$]$_n$ (1).

Temperature	298 K
Formula	$C_{10}H_6Cl_6N_2Zn$
Formula Weight	432.26
Crystal System	Tetragonal
Space group	P-4b2
a/ Å	13.8212(6)
b/Å	13.8212(6)
c/ Å	3.6510(2)
α/°	90
β/°	90
γ/°	90
V/ Å^3	697.43(7)
Z	2
D_c/ g cm^{-3}	2.058
μ /mm^{-1}	2.892
F(000)	424
θ range/°	2.9-25.6
Reflections collected	7836
Unique reflections	664
Reflections I > 2σ(I)	663
R_{int}	0.034
goodness-of-fit (F^2)	1.26
R1 (I > 2σ(I))	0.0167
wR2(I > 2σ(I))	0.0430
CCDC No.	1947282

Table S2. Selected Bond Lengths (Å) and Bond Angles (°) for 1.

**Bond Lengths**			
Zn1-Cl1	2.4885(4)	Zn1-Cl1^a	2.4885(4)
Zn1-Cl1^b	2.4885(4)	Zn1-Cl1^c	2.4885(4)
Zn1-N1	2.1886(19)	Zn1-N1^b	2.1886(19)
**Bond Angles**			
Cl1-Zn1-N1	90.00(4)	Cl1-Zn1-Cl1^a	94.38(1)
Cl1-Zn1-Cl1^b	85.62(1)	Cl1-Zn1-N1^b	90.00(4)
Cl1-Zn1-Cl1^c	180.00	Cl1^a-Zn1-N1	90.00(4)
Cl1^c-Zn1-N1^b	90.00(4)	N1-Zn1-N1^b	180.00
Cl1^b-Zn1-N1	90.00(4)	Cl1^a-Zn1-Cl1^b	180.00
Cl1^c-Zn1-N1	90.00(4)	Cl1^a-Zn1-Cl1^c	85.62(1)
Cl1^a-Zn1-N1^b	90.00(4)	Cl1^b-Zn1-Cl1^c	94.38(1)
Cl1^b-Zn1-N1^b	90.00(4)		

Symmetry code: a = x, y, 1+z; b =1-x, 2-y, z; c =1-x, 2-y, 1+z

S4. Characterization of Straight and Bent Crystal of $[Zn(\mu\text{-}Cl)_2(3,5\text{-dichloropyridine})_2]_n$ (1)

S4.1 Atomic Force Microscopy.

In order to establish the mechanical properties of CPs AFM force distance curves (FDC) was chosen a well-established method for such tasks.[16-17] For this method the AFM probe - which consist of a cantilever (spring constant k_c) and a microfabricated tip (radius R) - is brought into contact with the sample (approach) and withdrawn (retract) by means of a piezo. The applied force F is calculated from the cantilever deflection δ by Hook's law: $F = k_c\delta$, with k_c = 57.5 nN/nm. Deformation D is calculated from the difference of the piezo displacement Z and the cantilever deflection δ: $D = Z - \delta$.

With the tip radius R the geometry of the contact between probe and sample is defined and the reduced modulus E^* can be deduced from the deformation D in dependence of the applied force F by fitting the data with the Hertz theory (Eq 1)

$$D^{3/2} = \frac{F}{\sqrt{R}E^*} \qquad \text{(Eq 1)}$$

Knowing the mechanical properties of the AFM tip (E_{tip} = 170 GPa, ν_{tip} = 0.3) the Young's modulus of the sample can be calculated by Eq 2.

$$\frac{1}{E^*} = \frac{3}{4}\left(\frac{1-\nu_{tip}^2}{E_{tip}} + \frac{1-\nu^2}{E}\right) \qquad \text{(Eq 2)}$$

Reference measurements on a glass substrate were used for establishing the tip radius R. Since the mechanical properties of glass are well known (E_{glass} = 72 GPa, ν_{glass} = 0.3) the tip radius could be deduced from a fit with Eq 1 as R = 80 nm. Measurement (grey squares and grey line (inset)) and fit (black dotted line) are shown in Figure S4. All curves shown in Figure S4 and Figure S5 are averaged curves from at least 30 single curves, error bars are shown in Figure S4, inset.

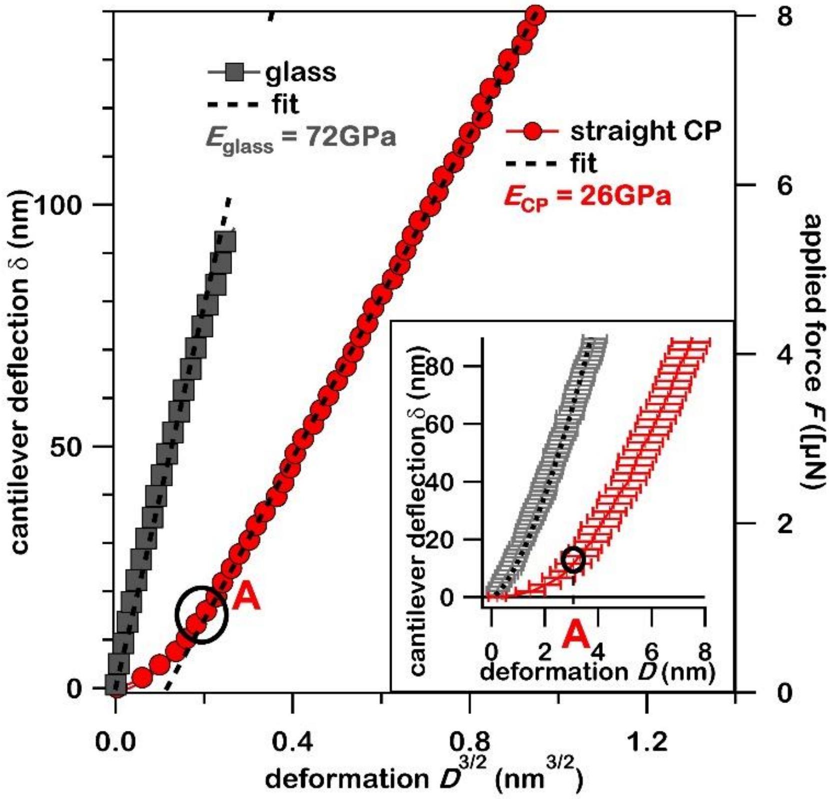

Figure S4. Main graph: deflection/force vs deformation$^{3/2}$ plot. The average glass curve (gray squares) with its fit by the Hertz theory is shown: in this plot the fit appears as a line (dotted line). The average curve of the straight CP is fitted as a mechanical double layer (dotted line) yielding the Young's modulus of bulk CP; point A shows the transition to the regime, where the properties of the bulk are dominant. **Inset:** deflection vs deformation plot of measurements of glass as a reference (grey) and CP (light red). Curves shown are average curves of at least 30 single curves (standard error is shown as error bar). The average glass curve is in accordance with the Hertz theory and was fitted by Eq 1 in order to ascertain the tip radius *R* (dotted line).

Straight crystals of (**1**) show high deformation rates directly following contact with the AFM tip (up to point A in Figure S4). This suggests the presence of a thin amorphous layer on the surface of (**1**) with thickness A (Figure S4). The thin amorphous layer on top of the crystal is considered a mechanical double layer as described by Silbernagl et al.[18-19] As is typical for mechanical double layers, the deformation $D^{3/2}$ plot shows a hyperbolic shape, which is a result of the mixed properties of the double layer. The expected hyperbolic shape is consistent with the plot shown in Figure S4, starting with a curvature transitioning into a linear section (at A in Figure S4), an approximation to an asymptotic behavior. The asymptotes of the hyperbola describe the deformation rates of the neat materials. The deformation induced up to the transition into a linear behavior (*D*=0 to A) corresponds to the thickness of the amorphous layer.

According to the theory of mechanical double layers the linear section of the $D^{3/2}$ in Figure S4 is consistent with the deformation rate of the bulk CP. A fit with Eq 1, as well as further calculations by Eq 2, yield a Young's modulus of E_{CP} = 26 GPa, with a Poisson's ratio of ν_{CP} = 0.28.

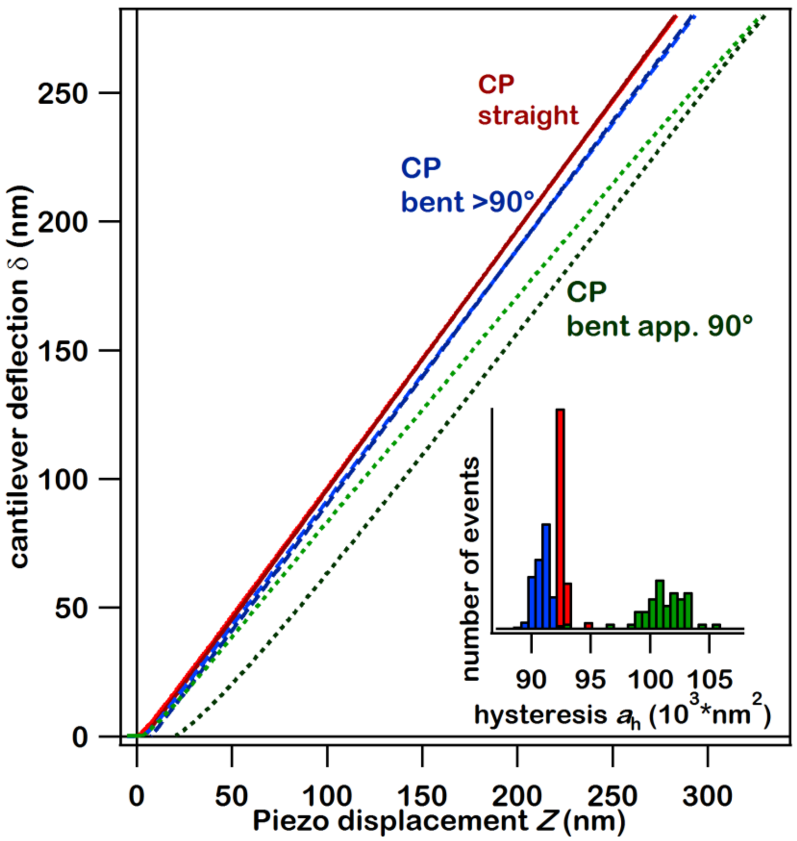

Figure S5. Deflection/force vs deformation plot of approach (load, red) and retract (unload, blue) curves. All curves shown are average curves of at least 30 single curves. Measurements of straight CP (red), obtuse bend CP (blue) and sharp bend CP (green) are compared. Inset: histogram of the hysteresis w_h calculated from single curves: straight CP (red) obtuse bend CP (blue) and sharp bend CP (green).

S4.2 Micro-focus single crystal X-ray diffraction

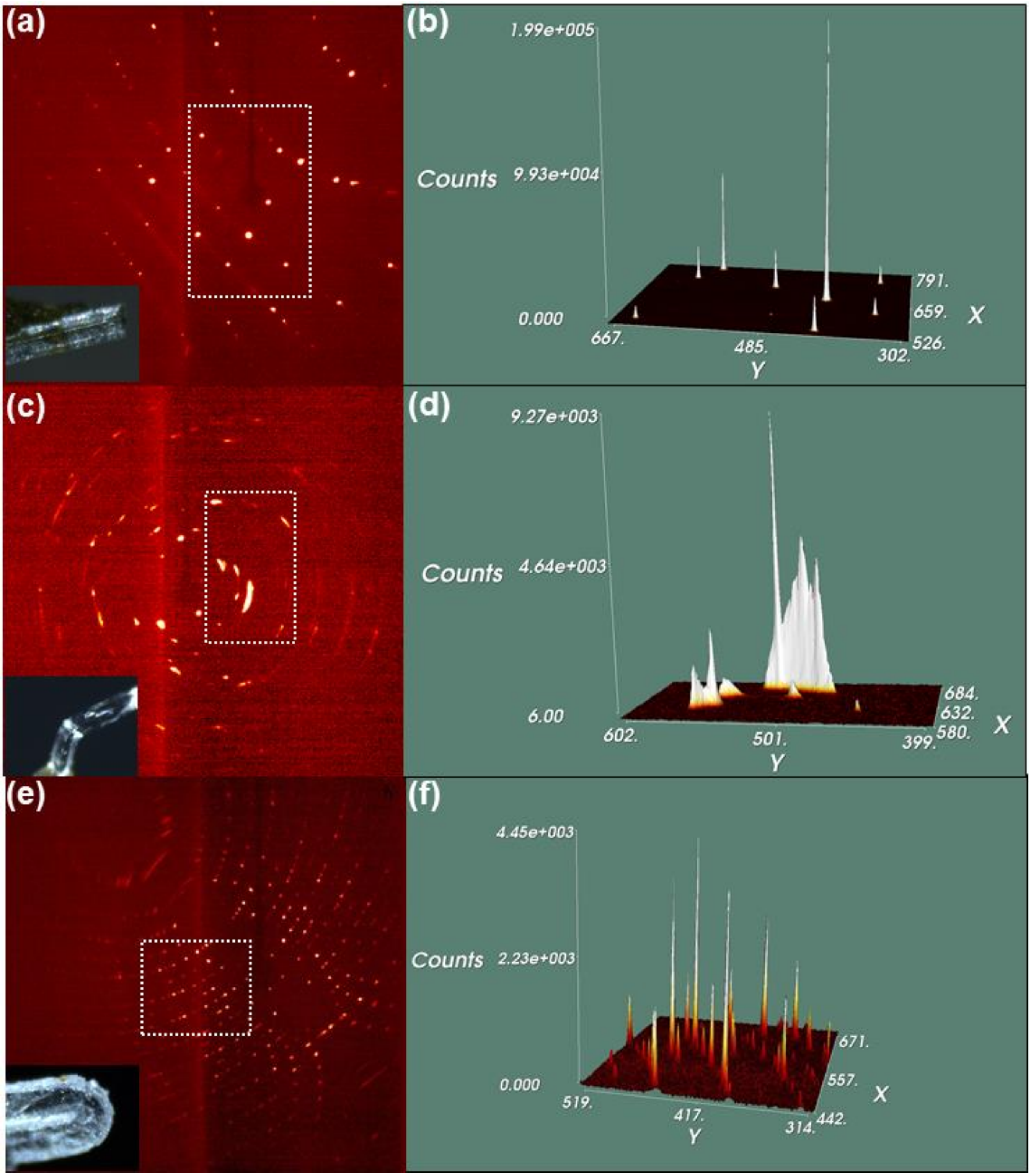

Figure S6. (a) Bruker diffraction image obtained from a straight crystal (inset). Normal shape with huge intensities of the diffraction spots from straight crystal; (b) 3D view of the area marked in (a). (c) Large elongation of the reflections from plastically bend crystal (> 90° bent; inset); (d) 3D view of the area marked in (c). (e) View of Debye-Scherrer rings from the reflection of highly bent crystal (<90° bent; inset); (f) 3D view of the area marked in (e).

S4.3 Raman spectroscopy

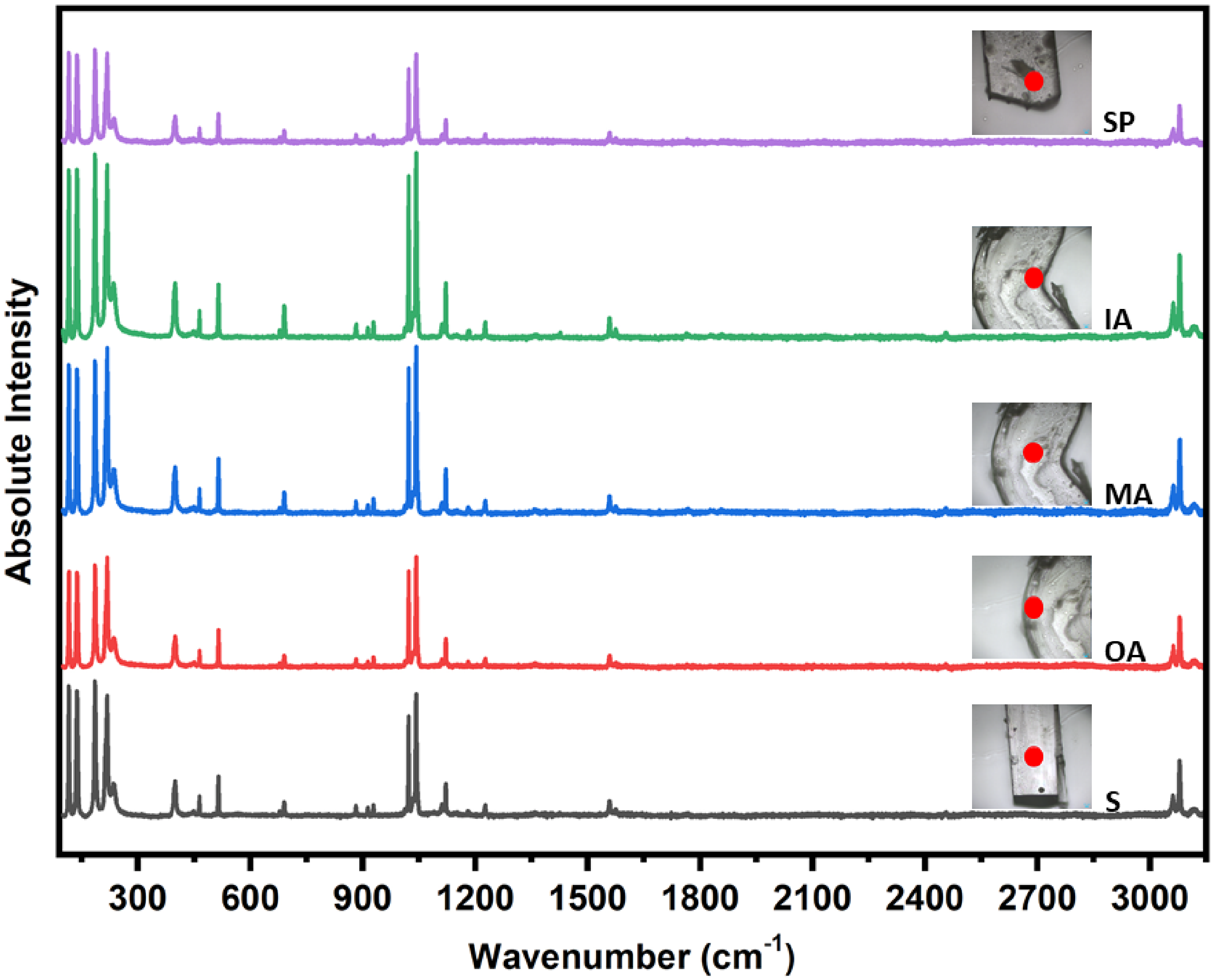

Figure S7. Unpolarised Raman spectra of [Zn(μ-Cl)$_2$(3,5-dichloropyridine)$_2$]$_n$ (**1**). Raman spectra were collected at different locations in the crystal at different stages of plastic bending. The Raman spectra are shown alongside the corresponding crystal, and the laser spot is indicated within a red dot. Crystals are denoted as s- straight; OA- outer arc; MA– middle arc; IA- inner arc; SP-straight part of bent crystal.

Almost no change in Raman band positions between straight and bent crystals was observed (see Figure 3 in the main text and Figure S7). The physical spectral resolution ranged from 0.5 cm^{-1} per CCD pixel to 0.3 cm^{-1} per CCD pixel within the observed spectral range of 100 cm^{-1} to 3800 cm^{-1}. Figure S8 and Table S3 summarise the results of peak fitting with Lorentz functions (using the software Origin from OriginLab), which was shown to improve the spectral resolution by approximately one order of magnitude.[20] No significant changes of wavenumber positions of 30 bands in each spectrum are observed, except for the signal at 235 cm^{-1}, which in the straight part of the bent crystal and in the straight crystal is at (235.65 ± 0.098) cm^{-1} and (235.60 ± 0.11) cm^{-1}, while in the bent part the band shifts from (235.06 ± 0.080) cm^{-1} (inner arc) via (235.78 ± 0.073) cm^{-1} (middle arc) up to (235.99 ± 0.079) cm^{-1} (outer arc).

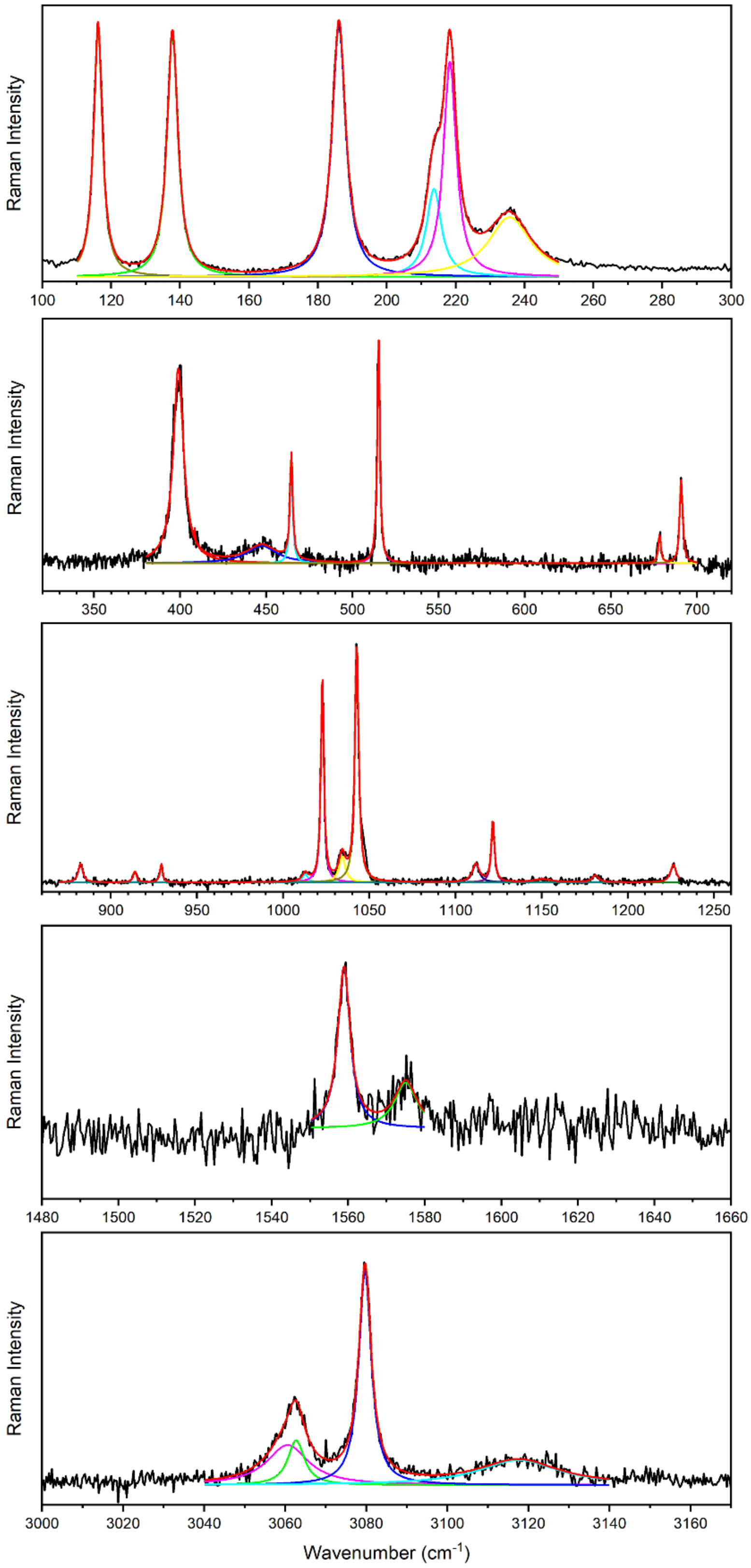

Figure S8. Peak analysis within the Raman spectrum SP (straight part of bent crystal), shown as an example. Within the investigated spectral range, 30 Raman bands were fitted with Lorentz functions using Origin (OriginLab). The results for all spectra are summarized in Table S3. Black is experimental data, pink, green, blue, and cyan are peaks deconvoluted from experimental data, and red is the sum of the deconvoluted peaks.

Table S3. Wavenumbers of 30 Raman bands in the samples SP, IA, MA, OA, and S (see Figure 3 in the main text) determined by Lorentzian peak fitting. Crystals are denoted as s- straight; OA- outer arc; MA– middle arc; IA- inner arc; SP-straight part of bent crystal.

SP (cm^{-1})	IA (cm^{-1})	MA (cm^{-1})	OA (cm^{-1})	S (cm^{-1})
**116.170** ± 0.0097	**116.110** ± 0.0085	**116.145** ± 0.0079	**116.269** ± 0.0088	**116.05** ± 0.012
**137.78** ± 0.011	**137.74** ± 0.010	**137.76** ± 0.010	**137.88** ± 0.010	**137.62** ± 0.014
**186.08** ± 0.012	**185.98** ± 0.010	**186.07** ± 0.010	**186.16** ± 0.011	**185.90** ± 0.015
**213.67** ± 0.063	**213.64** ± 0.058	**213.68** ± 0.051	**213.85** ± 0.054	**213.59** ± 0.077
**218.23** ± 0.022	**218.28** ± 0.021	**218.35** ± 0.016	**218.44** ± 0.017	**218.16** ± 0.025
**235.65** ± 0.098	**235.06** ± 0.080	**235.78** ± 0.073	**235.99** ± 0.079	**235.6** ± 0.11
**398.89** ± 0.044	**398.98** ± 0.071	**398.85** ± 0.032	**399.02** ± 0.034	**398.77** ± 0.049
**447.3** ± 0.90	**447.3** ± 0.53	**446.7** ± 0.71	**447.7** ± 0.68	**447.0** ± 0.98
**464.46** ± 0.042	**464.44** ± 0.033	**464.42** ± 0.033	**464.58** ± 0.033	**464.35** ± 0.049
**515.09** ± 0.019	**515.14** ± 0.015	**515.17** ± 0.013	**515.29** ± 0.013	**515.00** ± 0.017
**678.2** ± 0.15	**678.3** ± 0.10	**678.3** ± 0.16	**678.3** ± 0.12	**678.1** ± 0.24
**690.77** ± 0.053	**690.89** ± 0.031	**690.81** ± 0.042	**690.87** ± 0.049	**690.68** ± 0.068
**882.3** ± 0.14	**882.3** ± 0.13	**882.4** ± 0.14	**882.3** ± 0.15	**882.1** ± 0.19
**913.9** ± 0.21	**913.8** ± 0.14	**913.8** ± 0.16	**913.8** ± 0.19	**913.4** ± 0.14
**929.2** ± 0.11	**929.01** ± 0.088	**929.09** ± 0.084	**929.11** ± 0.084	**928.9** ± 0.10
**1012.7** ± 0.37	**1013.0** ± 0.20	**1013.0** ± 0.26	**1013.2** ± 0.27	**1012.8** ± 0.33
**1022.67** ± 0.011	**1022.692** ± 0.0083	**1022.724** ± 0.0083	**1022.788** ± 0.0087	**1022.72** ± 0.011
**1034.1** ± 0.12	**1034.2** ± 0.10	**1034.2** ± 0.11	**1034.3** ± 0.12	**1034.4** ± 0.15
**1042.64** ± 0.011	**1042.676** ± 0.0086	**1042.677** ± 0.0085	**1042.726** ± 0.0087	**1042.66** ± 0.011
**1111.8** ± 0.18	**1111.8** ± 0.17	**1111.8** ± 0.19	**1112.0** ± 0.19	**1111.8** ± 0.29
**1121.66** ± 0.037	**1121.61** ± 0.026	**1121.59** ± 0.028	**1121.70** ± 0.030	**1121.67** ± 0.038
**1150** ± 1.6	**1149** ± 1.6	**1150** ± 1.3	**1150** ± 1.7	**1148** ± 1.5
**1181.2** ± 0.46	**1182.8** ± 0.31	**1181.6** ± 0.33	**1181.4** ± 0.24	**1181.3** ± 0.83
**1226.4** ± 0.17	**1226.3** ± 0.13	**1226.5** ± 0.15	**1226.5** ± 0.16	**1226.7** ± 0.17
**1558.89** ± 0.091	**1558.75** ± 0.045	**1558.89** ± 0.053	**1558.78** ± 0.077	**1558.6** ± 0.14
**1575.1** ± 0.44	**1575.1** ± 0.15	**1575.1** ± 0.23	**1575.0** ± 0.46	**1574** ± 1.1
**3060.6** ± 0.86	**3060.6** ± 0.47	**3061.1** ± 0.41	**3060.3** ± 0.64	**3060.6** ± 0.72
**3062.7** ± 0.23	**3062.9** ± 0.11	**3063.4** ± 0.14	**3062.6** ± 0.14	**3062.1** ± 0.12
**3079.67** ± 0.02	**3079.73** ± 0.011	**3079.72** ± 0.014	**3079.81** ± 0.019	**3079.75** ± 0.026
**3117.6** ± 0.47	**3118.6** ± 0.20	**3118.2** ± 0.27	**3118.2** ± 0.36	**3119.3** ± 0.57

S5. Computational Vibrational Calculations

The calculated vibrational frequencies are given in comparison to experimentally collected Raman bands for the straight crystal, Table S4. In point group D_{2d} (-42m), normal modes with symmetry A_1, B_1, B_2, and E are Raman active. Group symmetry analysis shows that the primitive cell of [Zn(μ-Cl)$_2$(3,5-dichloropyridine)$_2$]$_n$ (**1**) contains a total of 150 normal modes, with $\Gamma_{acoustic} = B_2 + E$ and $\Gamma_{optical} = 16A_1 + 21A_2 + 16B_1 + 20B_2 + 37E$. Of the optical modes, $6Z - 3$ are external lattice modes (*Z*=2, for 9 external optical modes) and 138 optical molecular modes. With this symmetry analysis in mind, the large number of internal modes < 100 cm^{-1} does suggest it reasonable to expect some amalgamation of molecular modes into the lattice region. We note that the LO-TO splitting at $k \to 0$ was not calculated.

Overall, the low frequency modes are well captured within the PBE-D3 model, with discrepancies in experimental and calculated values differing on the order of < 3% below 1000 cm^{-1}. Discrepancy increases somewhat at higher wavenumbers, as is typical of the PBE DFT functional when handling molecular systems.

Table S4: Comparison of the calculated and experimental vibrational frequencies. Calculated values were obtained through finite differences at the PBE-D3 level. Experimental values were collected by microfocus Raman spectroscopy on a straight crystal at ambient temperatures.

SN.	Calculated (cm^{-1})	Irrep.	Exp. (cm^{-1})		SN.	Calculated (cm^{-1})	Irrep.	Exp. (cm^{-1})
1	26.944	A2	--		57	681.692	A1	--
2	28.839	B2	--		58	684.204	B1	--
3	38.754	E	--		59	684.925	E	690.68 ± 0.068
4	39.527	A2	--		60	808.992	B2	--
5	39.619	B1	--		61	812.676	E	--
6	44.095	E	--		62	817.547	A2	--
7	51.407	B2	--		63	867.488	B2	--
8	56.600	A1	--		64	868.357	A2	--
9	58.699	E	--		65	868.436	E	--
10	58.918	A2	--		66	891.164	A2	--
11	65.428	E	--		67	892.673	E	882.1 ± 0.19
12	85.021	A1	--		68	892.937	B2	--
13	98.522	A2	--		69	907.960	E	913.4 ± 0.14
14	99.098	B2	--		70	909.512	A1	928.9 ± 0.10
15	101.221	B1	--		71	909.714	B1	--
16	101.605	E	--		72	1015.317	B1	1012.8 ± 0.33
17	105.280	A2	--		73	1016.719	E	1022.72 ± 0.011
18	106.892	B2	--		74	1021.089	A1	1034.4 ± 0.15
19	119.810	E	116.05 ± 0.012		75	--	--	1042.66 ± 0.011
20	138.041	E	137.62 ± 0.014		76	1100.891	A2	--
21	141.312	B2	--		77	1103.559	E	--
22	143.063	A2	--		78	1104.086	B2	--
23	151.544	E	--		79	1109.229	E	1111.8 ± 0.29
24	162.612	A2	--		80	1111.667	B1	1121.67 ± 0.038
25	168.846	E	--		81	1116.670	A1	1148 ± 1.5
26	169.812	B2	--		82	1165.617	A1	--
27	187.367	E	185.90 ± 0.015		83	1166.237	E	--
28	187.844	B2	--		84	1169.508	B1	1181.3 ± 0.83
29	195.033	A2	--		85	1273.517	B2	1226.7 ± 0.17
30	199.651	A1	--		86	1276.589	E	--
31	201.173	B1	--		87	1276.663	A2	--

SN.	Calculated (cm^{-1})	Irrep.	Exp. (cm^{-1})		SN.	Calculated (cm^{-1})	Irrep.	Exp. (cm^{-1})
32	203.159	E	--		88	1307.749	B2	--
33	210.895	A1	--		89	1308.002	A2	--
34	210.936	E	--		90	1308.927	E	--
35	212.971	B1	213.59 ± 0.077		91	1405.579	B2	--
36	222.794	B1	218.16 ± 0.025		92	1407.828	E	--
37	230.559	E	235.6 ± 0.11		93	1410.211	E	--
38	231.656	A1	--		94	1410.438	B1	--
39	386.900	B2	--		95	1411.706	A1	--
40	387.048	A2	--		96	1411.728	A2	--
41	390.022	E	--		97	1543.686	B2	--
42	393.700	A1	--		98	1545.804	E	--
43	395.505	B1	--		99	1547.470	A2	--
44	395.706	E	398.77 ± 0.049		100	1559.688	B1	1558.6 ± 0.14
45	440.346	B2	447.0 ± 0.98		101	1560.350	E	1574 ± 1.1
46	441.600	E	--		102	1562.076	A1	--
47	442.539	A2	--		103	3139.117	E	3060.6 ± 0.72
48	461.775	A2	--		104	3139.147	A1	3062.1 ± 0.12
49	461.794	E	464.35 ± 0.049		105	3140.487	B1	3079.75 ± 0.026
50	462.496	B2	--		106	3141.432	B2	3119.3 ± 0.57
51	515.373	E	515.00 ± 0.017		107	3141.523	E	--
52	515.489	A1	--		108	3141.542	A2	--
53	515.889	B1	--		109	3144.659	E	--
54	673.673	B2	--		110	3144.915	A1	--
55	675.310	E	678.1 ± 0.24		111	3145.420	B1	--
56	675.929	A2	--					

In order to rationalize the vibrational spectrum of $[Zn(\mu\text{-}Cl)_2(3,5\text{-dichloropyridine})_2]_n$ (**1**), the eigenvectors were analyzed, and the eigenvectors for the vibrational coordinates at *ca.* (a) 40 cm^{-1} and (b) 50 cm^{-1} are shown in **Figure S9**. These vibrational modes correspond to (a) rotation of the chloropyridine ligands about the Zn-N bond, and (b) distortion about the Zn coordination sphere.

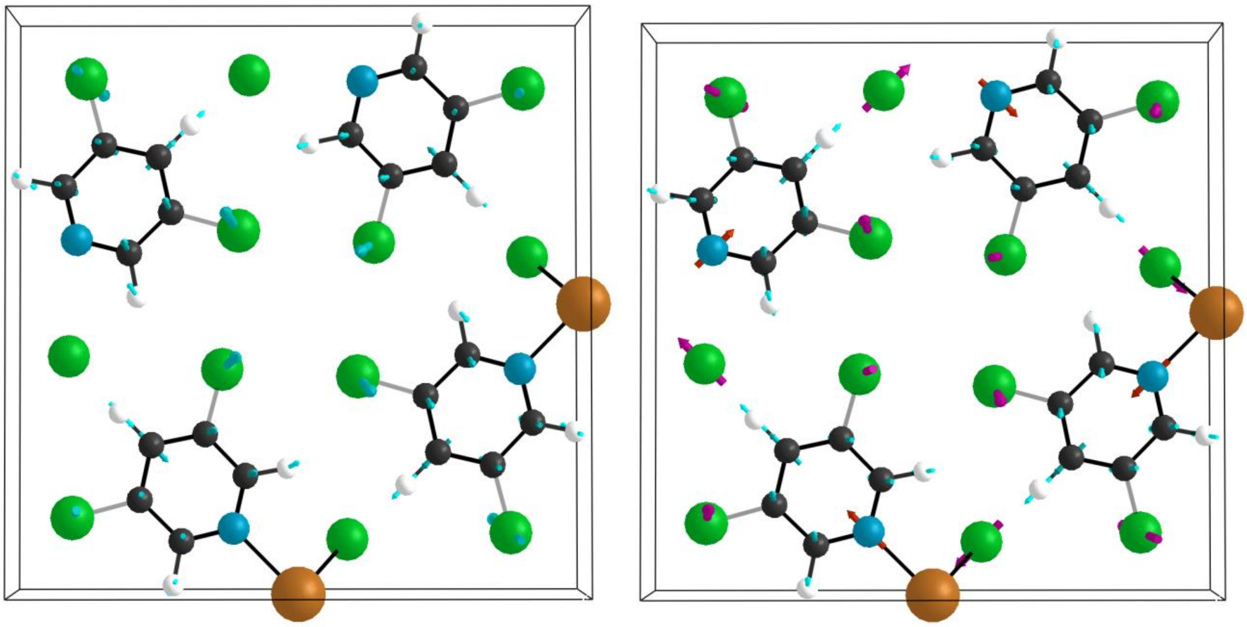

Figure S9. Visualisation of the Brillouin zone centre vibrational eigenvectors obtained at DFT-D3 level by finite differences. (Left) vibrational mode at 1.1877 THz (39.62 cm^{-1}). (Right) 1.6968 THz (56.60 cm^{-1}) Relative atomic displacements are indicated with arrows. Pink arrows indicate larger amplitudes. Atoms are coloured as (white) hydrogen, (black) carbon, (blue) nitrogen, (orange) zinc, (green) chlorine.

S6. Analysis of Coordination Polymer Potential Energy Surfaces

In order to rationalize the relative mechanical behavior of that [Zn(µ-Cl)$_2$(3,5-dichloropyridine)$_2$]$_n$, the potential energy surfaces (PES) associated with isotropic expansion of the unit cell along the crystallographic *a* and *b* axes (perpendicular to the CP chains) was obtained, Figure S10. This was compared with the corresponding PES of a previously reported elastically flexible CP network, [Cd(µ-Br)$_2$(2-chloropyrazine)$_2$]$_n$ (**2**).[21]

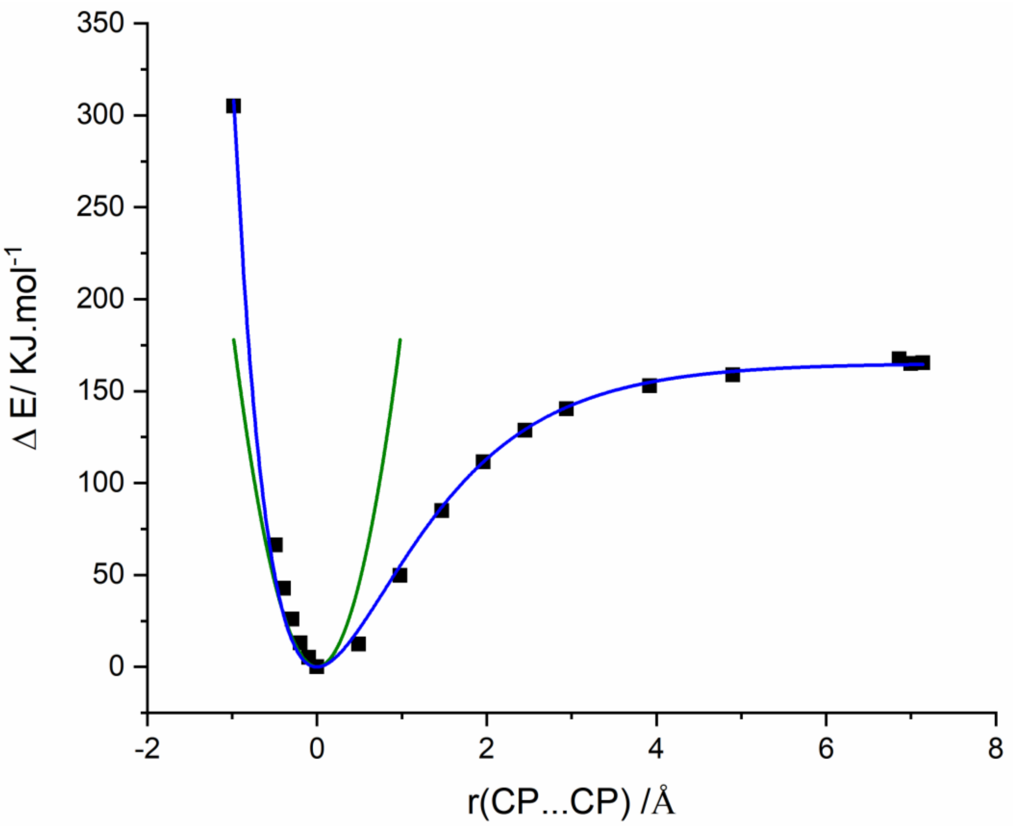

Figure S10: PBE-D3 Potential energy surface of [Zn(µ-Cl)$_2$(3,5-dichloropyridine)$_2$]$_n$ (**1**) associated with expansion of CP…CP interaction distance as a function of the metal-metal distances, r(CP…CP). Fits are exhibited for (blue) a conventional Morse potential, and (green) a harmonic potential.

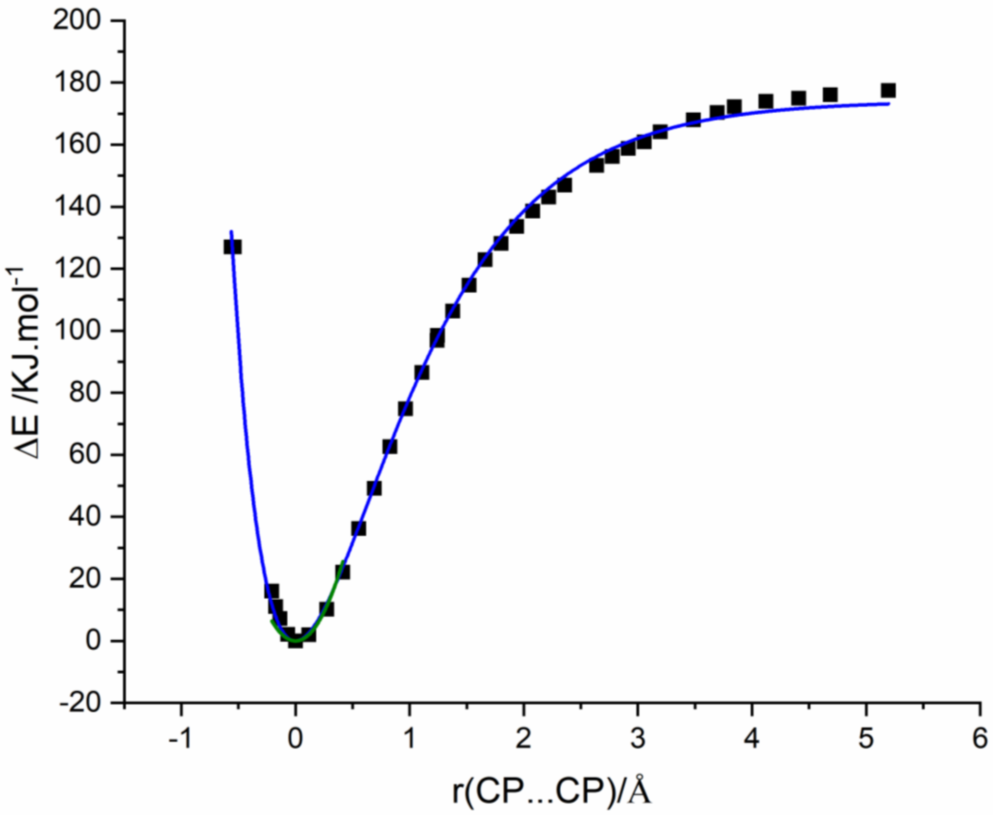

Figure S11: PBE-D3 Potential energy surface of [Cd(μ-Br)$_2$(2-chloropyrazine)$_2$]$_n$ (**2**) associated with expansion of CP…CP interaction distance as a function of the metal-metal distances, r(CP…CP). Fits are exhibited for (blue) a conventional Morse potential, and (green) a harmonic potential.

Table S5: Fitting data associated with the Morse and harmonic potentials in Figures S10 and S11.

**Potential Function**	**Force constant**	**Parameters plastic CP (1)**		**Parameters elastic CP (2)**	
$D_e \times \left(1 - exp\left(-a(X - R_e)\right)\right)^2$	$k = a^2 \times 2D_e$	D_e	165.18±3.06	D_e	174.21±0.90
		R_e	0 ±0	R_e	0 ±0
		a	0.88 ± 0.01	a	1.11 ±0.01
		k	255.01	k	430.82
$U = ½kx^2$	$k = \frac{d^2U}{dx^2}$	*k* = 370.89 ± 92.96		*k* = 298.83 ± 50.12	

S7. Qualitative Analysis of Electronic Structure

For a qualitative description of the interatomic (covalent) bonding interactions within the materials, we decomposed the charge density *via* the Crystal Overlap Hamilton Population (COHP). By plotting the negative value, we observe bonding interactions as positive, and anti-bonding interactions as negative. The COHP is projected onto select pairs of interacting atoms (pCOHP), thereby describing the interaction between the pair. The total integral of the pCOHP at the Fermi level is indicative of the strength of the covalent interaction.

When considering the structure of [Zn(μ-Cl)$_2$(3,5-dichloropyridine)$_2$]$_n$ (**1**), clear bonding interactions are observed between the Zn-Cl atoms, as expected. An integrated pCOHP of *ca.* 1 at the Fermi level gives clear indication of strong covalent character through the CP chain (Figure S12). As the Zn···Cl interactions form the backbone of the CP chain, it is not reasonable to expect facile rupture of the CP network upon bending (Figure S12). In contrast, projection of the COHP onto combinations of Cl···Cl interactions reveals no covalent interactions, with integrated pCOHP at the Fermi level < 0.02. While this does disprove the presence of electrostatic halogen bonding, it gives a clear indication that no orbital interactions are present.

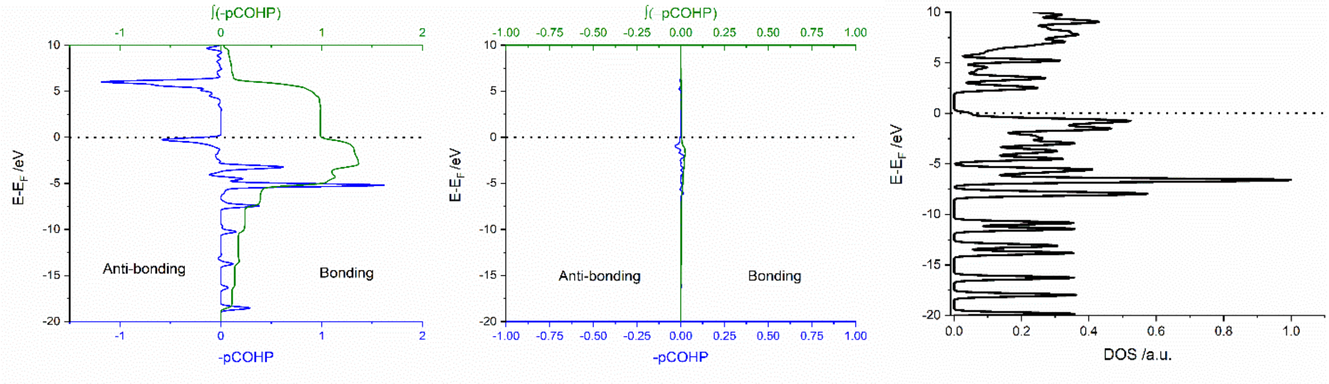

Figure S12. COHP analysis for [Zn(μ-Cl)$_2$(3,5-dichloropyridine)$_2$]$_n$ (**1**). (Left) COHP projected onto the Zn···Cl interactions. (Centre) COHP projected onto the Cl···Cl interactions. (Right) The total density of states (DOS) is given, normalised to unity.

The elastic CP, [Cd(μ-Br)$_2$(2-chloropyrazine)$_2$]$_n$ (**2**)[21] is constructed of Cd-Br interactions. The corresponding pCOHP is shown in Figure S13. The integral of the pCOHP value at the Fermi level is *ca.* 0.7, again demonstrating a strong covalent character through the CP chain. Hence again, it is unlikely that mechanical bending will lead to rupture of this interaction.

The structure of [Cd(μ-Br)$_2$(2-chloropyrazine)$_2$]$_n$ (**2**) contains a series of close intermolecular contacts *via* Cd···Br, and C-H···N pairs. For comparison to the structure of [Zn(μ-Cl)$_2$(3,5-dichloropyridine)$_2$]$_n$, we therefore consider the pCOHP of the halogen···halogen interactions, Figure S13. While the integrate pCOHP remains low at the Fermi level, it is an order of magnitude larger than in the plastic structure, suggesting some covalent character associated with the halogen···halogen interaction. Orbital character has been noted for relatively strong halogen···halogen interactions elsewhere.[22]

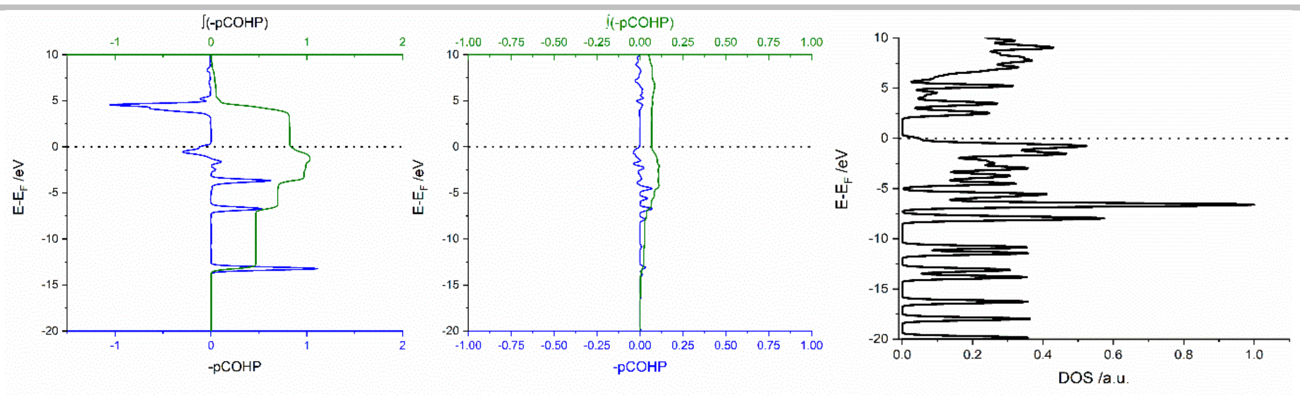

Figure S13. COHP analysis for $[CdCl_2(Cl\text{-}pyrazinamide)_2]_n$. (Left) COHP projected onto the Cd···Cl interactions. (Centre) COHP projected onto the Cl···Br interactions. (Right) The total density of states (DOS) is shown, normalised to unity.

While the pCOHP analysis as performed does not provide quantitative measure the atom-atom interactions present within the mechanically flexible CPs, it does clearly demonstrate that the CP network is composed of strong covalent bonding in each system. Furthermore, there is indication of strong halogen bonding interactions within the elastically flexible CP, which are absent from the plastic material.

S8. Intermolecular Interactions

As a rough indicator of the intermolecular interaction associated with the halogen bond in [Cd(μ-Br)$_2$(2-chloropyrazine)$_2$]$_n$, and [Zn(μ-Cl)$_2$(2-chloropyrazine)$_2$]$_n$ the electrostatic interaction was approximated according to

$$E_{Hal}^{Coul} = V(r)_{\sigma} \cdot q_X$$

where r is the interacting distance in the crystal structure, $V(r)_{\sigma}$ is the molecular electrostatic potential surface (MEPs) of the halogen σ-hold, and q_x is taken as the partial charge of the interacting atom, obtained by fitting MEPs *via* the CHELPG scheme. We note that this assumes close contact between the point charge and the MEP. The MEPs for both [Cd(μ-Br)$_2$(2-chloropyrazine)$_2$]$_n$, and [Zn(μ-Cl)$_2$(2-chloropyrazine)$_2$]$_n$ are given in Figure S14

This yields an approximate value of $\mathrm{E}_{\mathrm{Hal}}^{\mathrm{Coul}} \approx -0.203\ eV$ for the Cd…Br interaction of [Cd(μ-Br)$_2$(2-chloropyrazine)$_2$]$_n$ and $\mathrm{E}_{\mathrm{Hal}}^{\mathrm{Coul}} \approx +0.0044\ eV$ for the repulsive H…Cl interaction of Zn(μ-Cl)$_2$(2-chloropyrazine)$_2$]$_n$. Instead, accounting for the non-linear geometry of the H…Cl interaction, we map the negative V(r) to yield $\mathrm{E}_{\mathrm{Hal}}^{\mathrm{Coul}} \approx -0.013\ eV$

It is evident that the interaction in [Cd(μ-Br)$_2$(2-chloropyrazine)$_2$]$_n$ is substantially stronger than that in Zn(μ-Cl)$_2$(2-chloropyrazine)$_2$]$_n$.

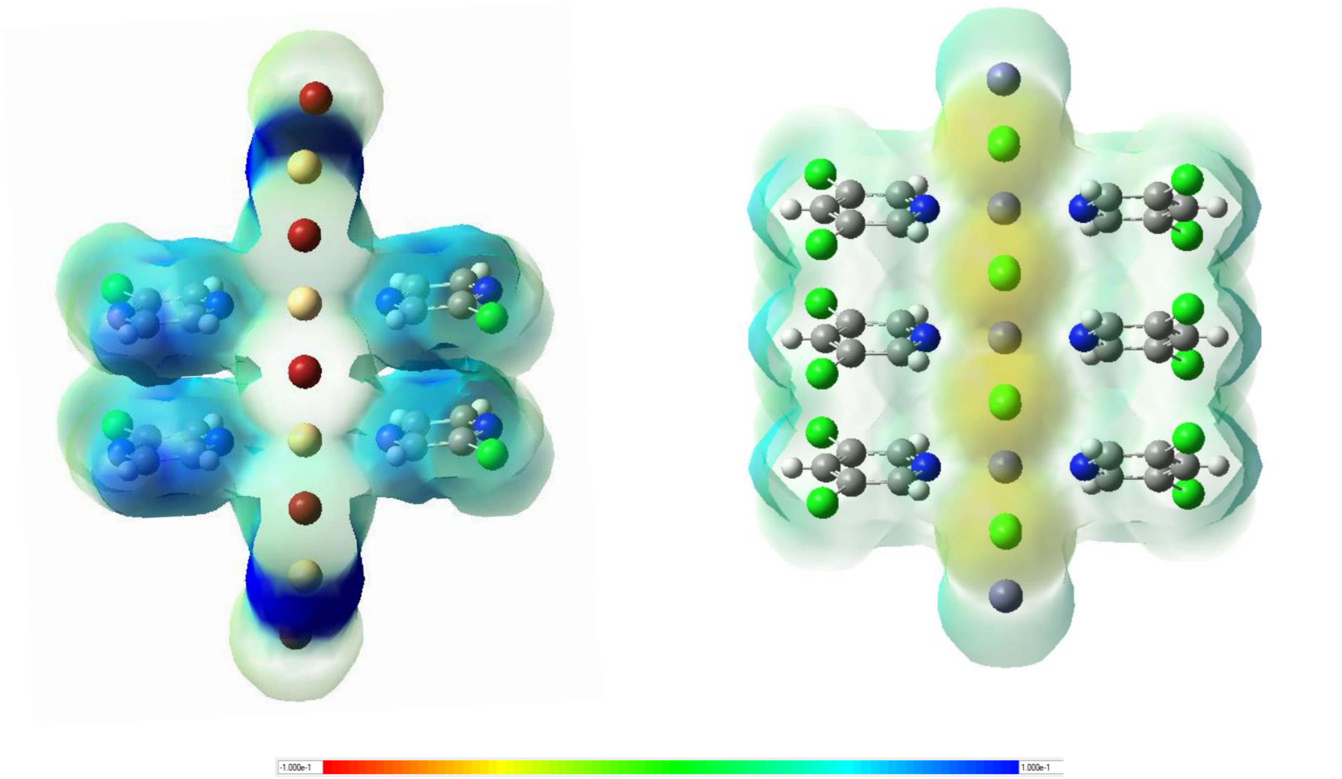

Figure S14. Molecular electrostatic potential for (left) [Cd(μ-Br)$_2$(2-chloropyrazine)$_2$]$_n$ (**2**) and (right) [Zn(μ-Cl)$_2$(2-chloropyrazine)$_2$]$_n$ (**1**) plotted with isosurface = 0.002 a.u. Atoms are coloured as (white) hydrogen, (light grey) carbon, (blue) nitrogen, (cream) cadmium, (maroon) bromine, (dark gray) zinc, (green) chlorine.

S9. PXRD and ATR-IR spectroscopy

S9.1 X-ray powder diffraction

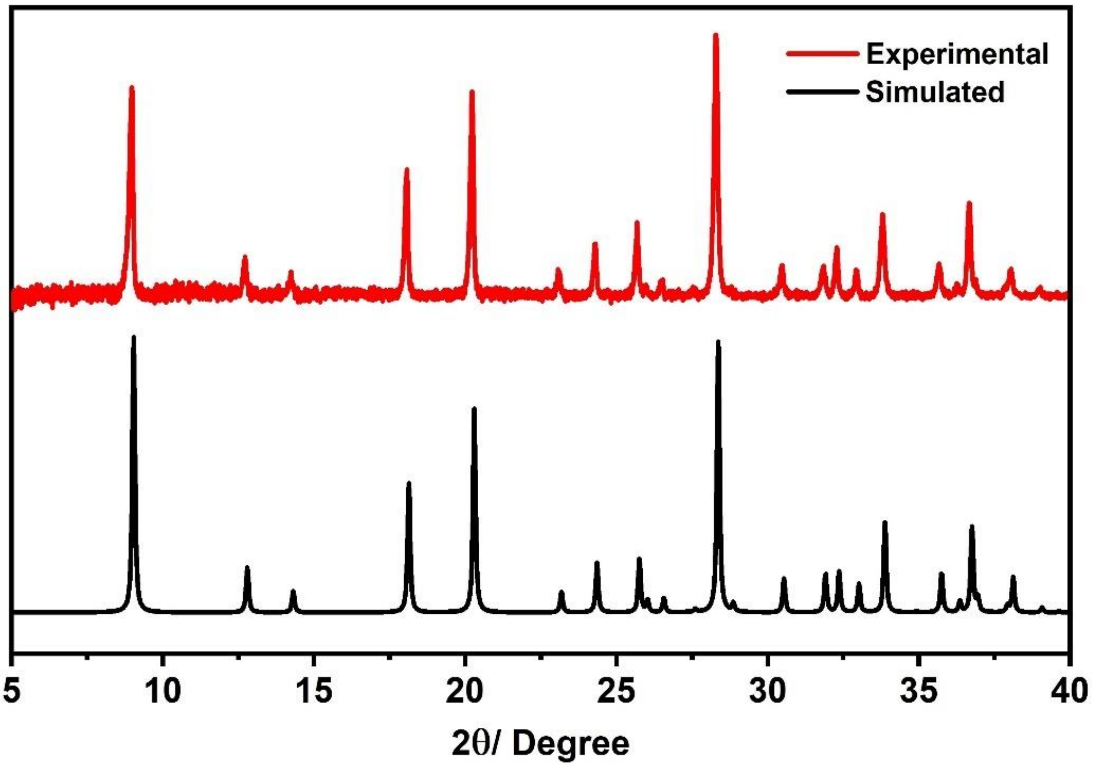

Figure S15. PXRD comparison of simulated pattern (black) and experimental pattern (red) for [Zn(μ-Cl)$_2$(3,5-dichloropyridine)$_2$]$_n$ (**1**).

S9.2 ATR-IR spectroscopy

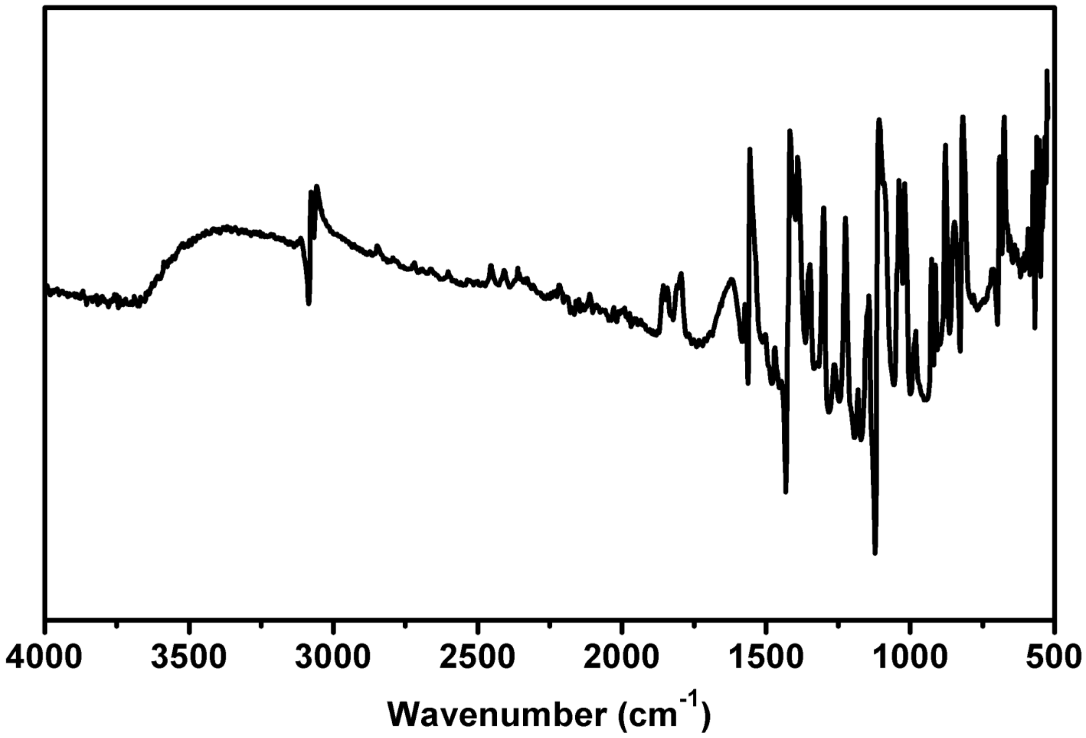

Figure S16. ATR-IR spectra of straight crystals of [Zn(μ-Cl)$_2$(3,5-dichloropyridine)$_2$]$_n$ (**1**).

S10. References

[1] SMART (V 5.628), SAINT (V 6.45a), XPREP, SHELXTL; Bruker AXS Inc.: Madison, WI, **2004**.

[2] G. M. Sheldrick, SADABS, Version 2.03; University of Gçttingen: Germany, **2002**.

[3] G. M. Sheldrick, *Acta Crystallogr. Sect. C* **2015**, *71*, 3–8.

[4] L. J. Farrugia, G. X. Win, *J. Appl. Crystallogr.* **1999**, *32*, 837–838.

[5] C. F. Macrae, P. R. Edgington, P. McCabe, E. Pidcock, G. P. Shields, R. Taylor, M. Towler, J. Van De Streek, *J. Appl. Crystallogr.* **2006**, *39*, 453–457.

[6] T. Schmid, P. Dariz, *Heritage* **2019**, *2*, 1662-1683.

[7] P. Giannozzi, S. Baroni, N. Bonini, M. Calandra, R. Car, C. Cavazzoni, D. Ceresoli, G. L. Chiarotti, M. Cococcioni, I. Dabo, A. Dal Corso, S. de Gironcoli, S. Fabris, G. Fratesi, R. Gebauer, U. Gerstmann, C. Gougoussis, A. Kokalj, M. Lazzeri, L. Martin-Samos, N. Marzari, F. Mauri, R. Mazzarello, S. Paolini, A. Pasquarello, L. Paulatto, C. Sbraccia, S. Scandolo, G. Sclauzero, A. P. Seitsonen, A. Smogunov, P. Umari, R. M. Wentzcovitch, *J. Phys.: Condens. Matter* **2009**, *21*, 395502.

[8] M. Đaković, M. Borovina, M. Pisačić, C. B. Aakeröy, Ž. Soldin, B. M. Kukovec, I. Kodrin, *Angew. Chem. Int. Ed.* **2018**, *57*, 14801–14805.

[9] J. P. Perdew, K. Burke, M. Ernzerhof, *Phys. Rev. Lett.* **1996**, *77*, 3865-3868.

[10] S. Grimme, J. Antony, S. Ehrlich, H. Krieg, *J. Chem. Phys.* **2010**, *132*, 154104.

[11] J. D. Pack, H. J. Monkhorst, *Phys. Rev. B: Solid State* **1977**, *13*, 5188–5192.

[12] A. Togo, I. Tanaka, *Scr. Mater.* **2015**, *108*, 1–5.

[13] Y. Hinuma, G. Pizzi, Y. Kumagai, F. Oba, I. Tanaka, *Comput. Mater. Sci.* **2017**, *128*, 140-184.

[14] S. Maintz, V. L. Deringer, A. L. Tchougréeff, R. Dronskowski, *J. Comput. Chem.*, **2016**, *37*, 1030–1035.

[15] F. Neese, *WIREs Comput. Mol. Sci.* **2012**, *2*, 73-78.

[16] H.-J. Butt, B. Cappella, M. Kappl, *Surf. Sci. Rep.* **2005**, *59*, 1–152.

[17] B. Cappella, S. K. Kaliappan, H. Sturm, *Macromolecules* **2005**, *38*, 1874–1881.

[18] D. Silbernagl, B. Cappella, *Surf. Sci.* **2009**, *603*, 2363–2369.

[19] D. Silbernagl, B. Cappella, *Scanning.* **2010**, *32*, 282–293.

[20] T. Schmid, R. Jungnickel, P. Dariz, *J Raman Spectrosc.* **2019**, *50*, 1154–1168.

[21] M. Đaković, M. Borovina, M. Pisačić, C. B. Aakeröy, Ž. Soldin, B. M. Kukovec, I. Kodrin, *Angew. Chem. Int. Ed.* **2018**, *57*, 14801–14805.

[22] M. P. Mitoraj, A. Michalak, *J. Mol. Model.* **2013**, *19*, 4681–4688.

Author Contributions

BB synthesized the crystals and performed macroscopic mechanical tests. AM and BP performed all the theoretical calculations. BB, MR and TF handled XRD and ATR-IR analysis. DS and HS handled AFM studies. TS performed micro-Raman spectroscopy. KR and AG performed Terahertz (THz) Time Domain (TD) spectroscopy. FE, BB and AM conceived the project and analysed results. FE supervised the work. FE, AM and BB wrote the manuscript with inputs from all co-authors.
